# Supplementary material for: Quantifying and preventing Plasmodium vivax recurrences in primaquine-untreated pregnant women: An observational and modeling study in Brazil
Source: PLoS Negl Trop Dis. 2020 Jul 31;14(7):e0008526. doi: 10.1371/journal.pntd.0008526 (PMC7423143; doi:10.1371/journal.pntd.0008526)
Supplement: S1 Text — Time-to-previous-episode analysis over 12 months of follow-up in PQ-untreated pregnant women and PQ-treated non-pregnant control experiencing a baseline vivax malaria episode. (PDF) [file pntd.0008526.s003.pdf]

Time-to-previous-episode

In order to verify the assumption that our study population displays a uniform risk of new infections, we analyzed the time-to-previous-episode over 12 months, starting with the baseline malaria episode for PQ-untreated pregnant women and PQ-treated non-pregnant control. Overall, 21 (13.3%) of 158 women who were pregnant at the baseline and 31 (9.8%) of 316 matched controls had one or more laboratory-confirmed *P. vivax* episode diagnosed within 12 months before their baseline vivax malaria episode. Participants with incident *P. falciparum* infection were censored at the time of diagnosis. Kaplan-Meier analysis show no statistically significant difference in the time to previous event (Log-rank test, p = 0.2; S1 Fig). Therefore, women who will become pregnant and their non-pregnant controls do not differ in their rates of new infections *before the baseline.*

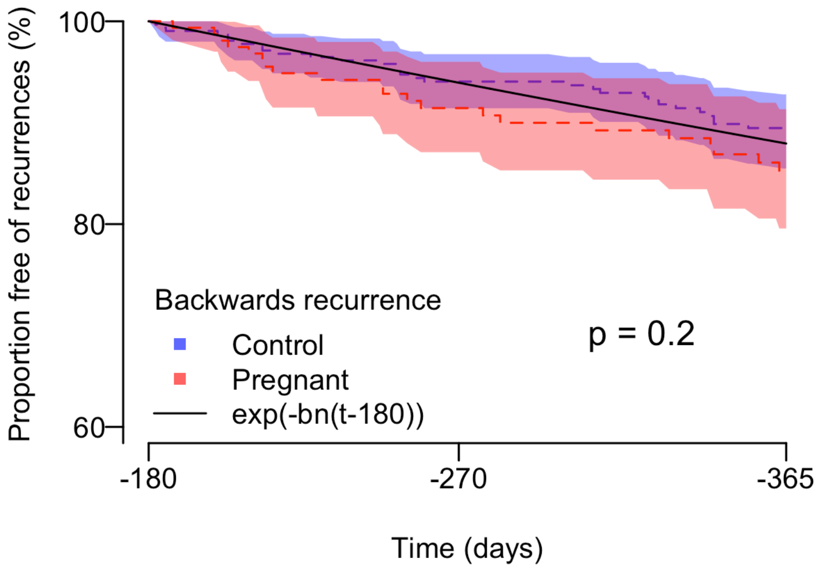

S1 Fig: Time-to-previous-episode over 12 months of follow-up in PQ-untreated pregnant women and PQ-treated non-pregnant control experiencing a baseline vivax malaria episode and exponential decay with rate β_n found in the time-to-next-event analysis. The shaded areas indicate the 95% confidence bands.

Even more importantly, survival function estimates obtained with the exponential model describing the slow dynamics of infection (β_n: rate of new infections) *after the baseline* are within the confidence bands of the Kaplan-Meier estimates obtained *before* the

baseline. We thus show that the β_n parameter inferred by fitting the model to prospective data (time to the next event; Fig. 3, main text) can properly describe the rate of new infections before the baseline (time to the previous event), when the vast majority of our "pregnant cases" were not yet pregnant. These findings support the assumption that our β_n estimate can properly describe the rate of new infections in both pregnant and non-pregnant women after and before the baseline.
