# Supplementary material for: Quantifying and preventing Plasmodium vivax recurrences in primaquine-untreated pregnant women: An observational and modeling study in Brazil
Source: PLoS Negl Trop Dis. 2020 Jul 31;14(7):e0008526. doi: 10.1371/journal.pntd.0008526 (PMC7423143; doi:10.1371/journal.pntd.0008526)
Supplement: Attachment — Submitted filename: PNTD-D-19-01699R1 abstract remodeled by DE gm resent June 24 2020.docx [file pntd.0008526.s004.docx]

PNTD-D-19-01699R1 please indeed here is the abstract June 24^th^ gm

Quantifying and preventing *Plasmodium vivax* recurrences in primaquine-untreated pregnant women: an observational and modeling study in Brazil

**In Latin America and the Caribbean, each year, 4.3 million pregnant women are exposed to *Plasmodium/P.* spp delivered by blood-feeding *Anopheles* hosting *P, vivax* or *P. falciparum* mature sporozoites in their salivary glands.** *Plasmodium vivax* causes 76% of the regional malaria burden and appears to be less affected than *P. falciparum* by current “elimination” efforts.

This is in part due to the parasite's ability to stay dormant in the liver and originate relapses within months after a single *Anopheles* inoculation.

Primaquine (PQ) is routinely combined with chloroquine (CQ) or other schizontocidal drugs to **suppress** *P. vivax* relapses and reduce the risk of late blood-stage recrudescence of parasites with low-grade CQ resistance. However, PQ is contraindicated for pregnant women, who remain at increased risk of repeated blood stage recrudescence **or mature sporozoites’ *P. vivax* re-exposure following CQ-only treatment.**

Here we apply a mathematical model to time-to-recurrence data from Juruá Valley, Brazil's main *Plasmodium* transmission hotspot, to quantify the extra burden of parasite recurrences attributable to PQ ineligibility in pregnant women. The model accounts for competing risks, since relapses and late **recrudescence** (that may be at least partially prevented by PQ) and **new exposure** (that are not affected by PQ use) all contribute to recurrences.

We compare recurrence rates observed after **primary *P. vivax-* driven parasitism** outcomes in 158 pregnant women treated with CQ only and 316 ***P. vivax* -driven parasitism outcomes** in non-pregnant control women, matched for age, “date of infection”, and place of residence, who were administered a standard CQ-PQ combination. We estimate that, once **hosting** *P. vivax,* 23% of the pregnant women have one or more “*vivax”* malaria recurrences over the next 12 weeks; 86% of these early *P. vivax* recurrences are attributable to relapses or late **recrudescence,** rather than **re-exposure to *P. vivax* mature sporozoites** that could be prevented by reducing exposure to Anopheline mosquitoes, during pregnancy.

Model simulations indicate that weekly CQ chemoprophylaxis extending over 4 to 12 weeks, starting after the first vivax malaria episode diagnosed in pregnancy, might reduce the risk of *P. vivax* recurrences over the next 12 months by 20% to 65%. We conclude that post-treatment CQ prophylaxis could be further explored as a measure to prevent “*vivax* malaria” recurrences in pregnancy and avert their adverse effects on **both maternal and neonatal Health.**
